# Knowledge of reproductive physiology and hormone therapy in 40-60 year old women: a population-based study in Yazd, Iran 

**Published:** 2012-07

**Authors:** Hossein Fallahzadeh, Maryam Hossienzadeh, Fatemeh Yazdani, Atefeh Javadi

**Affiliations:** 1 *Department of Biostatistics and Epidemiology, Health School, Shahid Sadoughi University of Medical Sciences, Yazd, Iran.*; 2 *Department of Public Health, Shahid Sadoughi University of Medical Sciences, Yazd, Iran.*

**Keywords:** *Menopause*, *Knowledge*, *Hormone therapy*, *Reproduction physiology*, *Women*, *Iranian*

## Abstract

**Background** **:** Evidences shows that menopause affects women's health, but women's knowledge of proper care and maintenance is insufficient.

**Objective:** To determine knowledge of hormone therapy (HT), reproductive physiology, and menopause in a population of 40-60 year old women.

**Materials and Methods:** This cross-sectional study was conducted through a cluster sampling among 330 women in Yazd, Islamic Republic of Iran, in 2010. Data was collected using a questionnaire containing questions about reproductive physiology related to menopause and HT by interviewing. Inferential and descriptive statistics via SPSS.15 software were used for data analysis.

**Results:** Overall, 2.1% of women were current takers of HT, 13.4% had taken it in the past but had stopped and 84.5% had never taken hormone replacement therapy. Iranian women had low knowledge of HT, reproductive physiology, and menopause. Most of the women (85.5%) knew that hot flashes are common around menopause and only 77.2% knew decreasing estrogen production causes the menopause. They knew little about the effects of progestagens and the effects of HT on fertility. Logistic regression determined that age, educational level and BMI were the most important factors predicting use of HT after adjusting for other variables.

**Conclusion:** Iranian women have a low HT usage rate and the majority of them are lacking of the knowledge about HT and menopause. Women need improved knowledge of the risks and benefits of HT as well as education about the reproductive system around menopause.

## Introduction

Physiological event that occurs with ovarian failure and marks the end of women's reproductive life is Menopause (1). 

Hormone replacement therapy (HRT) is an effective treatment for menopausal symptoms and can protect women from developing osteoporosis, although effectiveness is associated with continued use (2, 3). Yet with the publication of findings from studies such as the Heart and Estrogen/Progestin Replacement Study (4), the Women’s Health Initiative (WHI) (5), and the Million Women Study (6), the assumptions underlying the putative effects of HRT (in particular, estrogen plus progestin therapy) have undergone a stringent paradigm shift, with radical public perception changes reflected in altered prescribing practices (7).

A study of HRT and menopause in Europe has revealed that while women appear to be well-informed regarding the issues (83%), 40-50% reported having negative feelings towards HRT, despite the high prevalence of postmenopausal symptoms (8). Whereas some women opted for natural remedies, the majority did not opt for HRT because of the risks (breast cancer and side effects). 

Of the few post-WHI studies conducted in Asia, three recent retrospective studies evaluating changes in prescription use of HRT in Taiwan reported a pronounced decline in use (9), with college-educated women less likely to be prescribed HRT compared to lesser educated women (10). In a large study in five countries in Asia Huang KE *et al* reported that, there was a general lack of knowledge among these women regarding treatment options, HRT, and possible risks associated with HRT (11).

In Iran, there are only a few reports of the menopausal symptoms and knowledge about HRT in natural menopause women. A survey 4 years ago showed that The Iranian women demonstrated a moderate to high frequency of reporting four symptoms in vasomotor, vaginal, sleep-related, emotional, and somatic categories (12). In another study in south of Iran showed less than 7% of women knew that HRT could be used to treat the menopause, despite the high prevalence of postmenopausal symptoms (13). In order to meeting the needs and demands of menopausal women, it is important that physicians and other healthcare providers be able to give the women correct information about the advantages and disadvantages of the therapy and alternatives, including lack of therapy compliance.

This information must be presented in a way that considers a woman's knowledge of HT and the menopausal transition and also her own reproductive functions. As far as we know, there are very few reports about women’s knowledge of female reproductive physiology related to menopause while numerous evidences are available about women’s knowledge of the risks and benefits associated with HT. 

The aim of this study was to survey to investigate the knowledge of HT, reproductive physiology, and menopause in a population of Iranian women 40-60 years old. We then wanted to determine whether the knowledge held by users of HT differed from that of nonusers of HT.

## Materials and methods

This cross-sectional survey was conducted between April 2010 and Jun 2010 in Yazd city. Yazd one of the large cities of the Islamic Republic of Iran, is the center of Yazd province. The city is located 750 Km south of the capital Tehran.

**Sample size and sampling method**

From previous studies, it is known that, 15% of post-menopausal women (13) were used HT and with marginal of error 4%. Thus, a total of 330 women were needed. Sampling was conducted based on the cluster method. Each cluster included 30 households' indifferent sections of the city. The statistical frame work used based on the household lists available in the department in Yazd province. 

At the first stage, the number of households for each area was accumulated, and then the sampling interval was computed. Women aged 40-60 years, who had lived in Yazd, were included in this study. A questionnaire regarding women's knowledge of the reproductive physiology, menopause, and HT was used. After each question, women were asked to check one of a number of boxes, one of which always related to the alternative "I do not know". 

The questionnaire was developed from a questionnaire originally in several steps and used in 1999 in a similar population (17-18). An almost identical version was used in 2003 and 2007 with the addition of a section about knowledge of reproductive physiology (19). A Persian version of the questionnaire was sent to 30 women, 50-60 years of age. The questionnaire was tested for validity by two experts, and reliability was tested by cronbach’s Alpha (r=0.78). The sociodemographic data included the marital status, the education level, the occupation, the number of children, the Wight the high, and the monthly family income. 

Reproductive characteristics included an assessment of menopausal status which, was defined as the following: (a) women who had regular menstrual periods in the last 3 months were classified as premenopausal, (b) women who indicated their periods had become irregular but they had a period in the last 12 months were classified as perimenopausal, (c) women who indicated they had not had a period in the last 12 months or longer were classified as postmenopausal.

**Statistical analysis**

The data analyzed using the statistical package SPSS version (15.0). Descriptive statistics were used where appropriate. The associations between the HT-use and sample characteristics were examined using chi-square test. Multivariate logistic regression was used to identify potential indicators of current HT-use.

## Results

Of the 330 respondents, 138 (41.8%) were pre-menopausal or Peri-menopause and 192 (58.2%) post-menopausal. Seven of the women (2.1%) were currently taking hormone replacement therapy, 44 (13.4%) had taken it in the past but had stopped and 279 (84.5%) had never taken hormone replacement therapy. The average duration of hormone use was 14.2±22 months. Mean age of respondents was 50.78 (SD=7.11).

The percentages of ever-users of HT were different in the different educational groups (Illiterate=5%, primary=20.6%, Intermediate= 2.4%, diploma=17.9%, university=14.8%) Educational level, BMI and other demographic data according to HT use are presented in table I. There were a significant relation between age and employment with HT use. 

The results of the questions on women's knowledge of menopause and reproductive physiology in relation to HT use are summarized in table II. 

Most of the women were aware that it is common for menopausal women to experience hot flashes (ever HT use=92.2%, Never HT use=84.2%) and that the menopausal transition is caused by decreasing estrogen production (ever HT use=45.1%, never HT use=35.8). In the ever HT use group, almost 43% of the women agreed that menopausal women with distressing symptoms needs HT, compared with 34% in other groups (p=0.176).

Women's knowledge of the risks and benefits of HT was very low. A large proportion of women provided responses of "I don't know" to question regarding the association of HT with specific conditions. Only 41.2% of women who were using HT agree that HT may be increase the risk of breast cancer, compared with 15.8%, in never user group (p=0.0002).

About 37% of ever-users of HRT answered that HT is recommended of women older than 50 years age, compared 25.5% in other group (p=0.02). Between 23-39% of women in the different HT user groups were believe that HT need for postmenopausal women (p=0.02). About 41% of the ever users of HT answered that HT change the quality of life, and among never-users, compared 24% in never user group (p=0.02). 

Logistic regression analysis was performed to test the background variables (Table I) to determine the importance of each independent variable in its association with the HRT use. It revealed that there were significant associations between HT use with educational level, BMI and as age (Table III).

**Table I:** characteristics of the women and comparisons between ever and never users of hormone therapy (HT).

**Variable**			**Total** **n (%)**		**HT ever-user** **n (%)**	**HT never-user** **n (%)**	**p-value**
Menopause stage							
	Premenopausal or perimenopause			138(41.8)			
	Post-menopause			192(58.2)			
	HT						
		User (current/pervious)		51 (15.5)			
		Never-user		279 (84.5)			
	Education						
		Illiterate		40 (12.1)	2 (5)	38 (95)	0.016
		Primary		155 (47)	32 (20.6)	123 (79.4)	
		Intermediate		41 (12.4)	1 (2.4)	40 (97.6)	
		Diploma		67 (20.3)	12 (17.9)	55 (82.1)	
		University		27 (8.2)	4 (14.8)	23 (85.2)	
BMI (kg/m^2^)							
		<25		105 (31.8)	22 (2.1)	83 (79)	0.33
		25-29.9		139(42.1)	20 (14.4)	119 (85.6)	
		>30		82 (24.8)	8 (9.8)	74 (90.2)	
	
		Mean± SD		27.25±4.54			
Marital status							
		Single		10 (3)	1 (10)	9 (90)	0.262
		Married		281 (85.2)	40 (14.2)	241 (85.8)	
		Divorced		6 (1.8)	2 (3.3)	4 (66.7)	
		Widowed		33 (10)	8 (29.2)	25 (78.8)	
Employment							
		Housewife		286 (86.7)	39 (13.6)	247 (86.4)	0.02
		Full time		44 (13.3)	12 (27.3)	32 (72.7)	
Monthly income							
		Low		61 (18.5)	6 (9.8)	55 (90.2)	0.392
		Medium		255 (77.3)	43 (16.9)	212 (83.2)	
		High		14 (4.2)	2 (14.3)	12 (87.7)	
Age of respondents							
		40-49		146 (55.1)	5 (3.4)	141 (96.6)	0.0001
		50-59		119 (44.9)	30 (25.2)	89 (74.8)	
		Mean± SD		50.78 ± 7.1			
Parity							
		0		54 (16.4)	6 (11.1)	48 (88.9)	0.603
		1-2		167 (51.2)	26 (15.6)	141 (84.4)	
		≥3		109 (33)	19 (17.4)	90 (82.6)	
		Mean± SD		3.9±1.67			

**Table II:** Frequency of answers to questions about knowledge of menopause and reproductive physiology in 40 to 60 years-old in relation to hormone therapy-use

**Question**	**HT use**	**Agree** **n (%)**	**Disagree** **n (%)**	**I don't know** **n (%)**	**p-value**
Menopausal women with distressing symptoms need HRT.					
	Ever HT use	22 (43.1)	11 (21.6)	18 (35.3)	0.176
	Never HT use	95 (34.1)	46 (16.5)	138 (49.5)	
	Total	117 (35.5)	57 (17.2)	156(47.3)	
HT may be increase the risk of osteoporosis.					
	Ever HT use	12 (23.5)	10 (19.6)	29 (56.9)	0.026
	Never HT use	55 (19.7)	23 (8.2)	201 (72)	
	Total	67 (20.3)	33 (10)	230(69.7)	
HT may be increase the risk of breast cancer					
	Ever HT use	21 (41.2)	5 (9.8)	25 (49)	0.0001
	Never HT use	44 (15.8)	15 (5.4)	220 (78.9)	
	Total	65 (19.6)	20 (6.1)	245(74.3)	
HT may be increase the risk of coronary heart disease					
	Ever HT use	12 (23.5)	2 (3.9)	37 (72.5)	0.013
	Never HT use	26 (9.3)	16 (5.7)	237 (84.9)	
	Total	38 (11.5)	18(5.5)	274(83)	
	**HT use**	**Yes** **n (%)**	**No ** **n (%)**	**I don't know** **n (%)**	
Is a postmenopausal woman who has regular bleeding due to HT fertile?					
	Ever HT use	3 (5.9)	22 (43.1)	26 (51)	0.091
	Never HT use	17 (6.1)	78 (28)	184 (65.9)	
	Total	20 (6)	100(30.3)	210 (63.7)	
Does HT work as an oral contraceptive to prevent pregnancy?					
	Ever HT use	4 (7.8)	18 (35.2)	29 (56.9)	0.253
	Never HT use	22 (7.9)	68 (24.4)	189 (67.7)	
	Total	26 (7.9)	86 (26.1)	218 (66)	
Doses HT change the quality of life?					
	Ever HT use	21 (41.2)	10 (19.6)	20 (39.2)	0.001
	Never HT use	78 (28)	22 (7.9)	179 (64.2)	
	Total	99 (30)	32 (9.7)	199(60.3)	
HT is recommended to all women older than 50 years age					
	Ever HT use	19 (37.3)	13 (25.5)	19 (37.3)	0.02
	Never HT use	69(24.7)	47 (16.8)	163 (58.4)	
	Total				
Do you believe HT need for postmenopausal women?					
	Ever HT use	20 (39.2)	12 (23.5)	19 (37.3)	0.026
	Never HT use	72 (25.8)	46 (16.5)	161 (57.7)	
	Total	92 (27.9)	58 (17.6)	180 (54.5)	
Is it positive view point's your wife to HT?					
	Ever HT use	8 (15.7)	1 (2)	42 (82.4)	0.0001
	Never HT use	7 (2.5)	20 (7.2)	252 (90.3)	
	Total	15 (4.5)	21 (6.4)	294(89.1)	
Is menopause caused by decreasing estrogen production?					
	Ever HT use	23 (45.1)	1 (2)	27 (52.9)	0.287
	Never HT use	100 (35.8)	2 (0.7)	177 (63.4)	
	Total	123 (37.2)	3 (1)	204 (61.8)	
Why does menstrual bleeding case?					
	Ever HT use	11 (21.6)	6 (11.8)	34 (66.7)	0.788
	Never HT use	72 (25.8)	28 (10)	179 (61.5)	
	Total	83 (25.2)	34 (10.3)	213 (64.5)	
Women usually experience hot flashes and sweating during the menopause.					
	Ever HT use	47 (92.2)	2 (3.9)	2 (3.9)	0.093
	Never HT use	235 (84.2)	5 (1.8)	39 (14)	
	Total	282 (85.5)	7 (2.1)	41 (12.4)	

**Table III:** The association of background variables with HT use; multivariate analysis

**Variables**		**OR**	**P-value**	**CI**
Age (years)			0.02	
	40-49	1		
	50-59	8.03	0.0001	2.95-21.84
Educational level			0.049	
	Illiterate	1		
	Primary	5.98	0.026	1.23-27.81
	Intermediate	1.04	0.937	0.08-13.69
	Diploma	3.55	0.166	0.59-21.03
	University	1.34	0.80	0.14-13.25
Marital status			0.71	
	Single	1		
	Married	1.4	0.767	0.15-13.04
	Divorced	1.26	0.887	0.05-32.12
	Widowed	3.06	0.365	0.28-34.53
BMI (kg/m^2^)			0.06	
	<25	1		
	25-29.9	0.52	0.092	0.25-1.10
	>30	0.31	0.028	0.12-0.80
Parity			0.092	
	0	1		
	1-2	1.07	0.9	0.36-3.23
	≥3	1.07	0.9	0.32-3.63
Employment			0.058	
	Housewife	1		
	Full time	3.45	0.058	0.96-9.8
Monthly income			0.394	
	Low	1		
	Medium	2.02	0.194	0.68-5.84
	High	2.89	0.32	0.35-23.32

## Discussion

Menopause marks a time of dramatic hormonal and often social change for women (20). When life expectancy is rising, more women are exposed to the potential long term consequences of menopause. HRT can have significant benefits in postmenopausal women, yet rates of HT use are low.

The present population-based study in Yazd, Iran showed that 40-60 year old women had rather limited and in our opinion insufficient knowledge of certain aspects of menopause and HT. The overall past and current rates of use of HT (15.1 and 2.1%, respectively), in our study were not only slightly lower than Asian countries including Hong Kong, Taiwan, China and Japan population (21-24), but also much lower than USA and countries in Europe such as Swedish, Germany and England (19, 25-27). 

Probably the low rate of HT use in Iranian population is related to problem of compliance rather than the low demand. Previous studies showed that prevalence of vasomotor symptoms in this area was very high (12). HT use was different among women with different educational level. Previous studies (19, 28) have shown that HT use was more common in women with higher education than women with lower education, but this relation seems to be declining (19, 28, 29). 

More than 50% of current or previous HT users were unsure about the effect of menopause on decreasing estrogen production. Thus, knowledge of hormonal effects on the reproductive organs is low among women and the information given by healthcare providers about effect of estrogens and progestagens seems either to be in sufficient. It is notable that almost half of the women in ever HT user group and 65% of women in never HT user group did not know that recurrence of bleeding during HT in post-menopausal women does not mean that there is a risk of pregnancy. 

This may lead to think that contraception should be used if bleeding returns when using HT, and this may hamper their sex, life of even cause them to abstain. Only about 40% women knew that menopausal transition is used by decreasing estrogen level, but most of them knew that hot flashes are common in the menopausal transition. In previous studies, Buick *et al* (29) and Astrand *et al* (19) reported a great variation in women's knowledge of cause of menopause, only 11% of UK women knew the cause, compared with 80% in Sweden.

Nearly 70% of the women in study performed in Chile in 2001 stated that they had insufficient knowledge of HT and menopause (14). We have found few studies on women's knowledge of reproductive physiology. This study showed that women in general have not good knowledge of the risks and benefits of HT concerning osteoporosis and coronary heart disease. For example, only 41% of the women (in ever HT user group) knew that the risk of developing breast cancer increased when using HT. Most of women were uncertain about the risk of developing myocardial in fraction when using HT, This uncertainty may be a result of the exposure to contradictory findings during the past years, which have affected national treatment recommendations and have been frequently debated in the media. 

There may even be discussion of what the correct answer is this question because the risk-benefit profile differs depending on when HT in started, dose used, design and duration of the study (30). In a European study from 2003, the results showed, more than half of the women who were ever-users of HT being aware of the increased risk of breast cancer and one third of the women being uncertain about the risk of developing heart disease when using HT (15).

Swedish studies performed in 1998 and 2007 showed that 45% and 12% of the women, respectively, thought that the risk of developing cardiovascular disease decreased with HRT use (19, 31). In summary, this study showed that Iranian women have a low HT usage rate and insufficient knowledge of reproductive physiology and HT. This study demonstrate the need to target education on the use HT to younger, less educated, and housewife women providing better communication and discussion with healthcare providers could empower women to manage an important period of their lives. 

Random sampling and results generalizability to the real community are the strength our study. Low literacy level of participants and then incomplete understanding of questions was a limitation of the study.
